# Harm reduction in Cambodia: a disconnect between policy and practice

**DOI:** 10.1186/1477-7517-9-30

**Published:** 2012-07-09

**Authors:** Kannarath Chheng, Supheap Leang, Nick Thomson, Timothy Moore, Nick Crofts

**Affiliations:** 1 National Institute for Public Health, #2, Street 289, Toul Kork, Phnom Penh, Cambodia; 2 Nossal Institute for Global Health, University of Melbourne, Melbourne, Australia; 3 Centre for Law Enforcement and Public Health, 161 Barry Street, Carlton, 3010, Australia; 4 Johns Hopkins Bloomberg School of Public Health, Baltimore, USA; 5 Melbourne School of Population Health, University of Melbourne, 207 Bouverie Street, Parkville, 3100, Australia

## Abstract

In 2003 the Government of Cambodia officially began to recognise that harm reduction was an essential approach to preventing HIV among people who use drugs and their sexual partners. Several programs aiming to control and prevent HIV among drug users have been implemented in Cambodia, mostly in the capital, Phnom Penh. However, there have been ongoing tensions between law enforcement and harm reduction actors, despite several advocacy efforts targeting law enforcement. This study attempts to better understand the implementation of harm reduction in Cambodia and how the policy environment and harm reduction program implementation has intersected with the role of law enforcement officials in Cambodia.

## Introduction

Cambodia is a country of about 14 million people, sharing land borders with Lao PDR, Thailand and Vietnam [1]. Despite recent economic growth, Cambodia remains one of the poorest countries in the region: the country’s GDP per capita stood at $830 in 2010, and the proportion of people living under the poverty line was about 30% of the population in 2007 [2,3]. Cambodia’s geographical location, poverty, high level of illiteracy and the loose legal system resulting from decades of internal conflicts have made Cambodia especially vulnerable to drug trafficking, drug use and its consequences [4].

Estimations of the availability, patterns of use and trends of illicit drug use in Cambodia vary. In 2004, an expert consensus group estimated there to be 20,000 Amphetamine-Type-Stimulant (ATS) users and 2,500 heroin users, of who about 1,750 inject. In 2007 the National Authority for Combating Drugs (NACD) reported that there were 5,797 drug users who had come into contact with local authorities, during the same time period UNAIDS estimated that there were 46,300 drug users in the country of whom 23,150 (50%) were using ATS and some 2,900 (6.3%) heroin, including 2,025 (range: 1,250-7,500) who injected [5]. The UNODC has reportedly estimated that 4% of Cambodia’s population have ever used drugs which would mean a potential estimation of the total number of drug users of around 500,000 [4] although the non-injecting use of ATS would account for the majority of this figure as ATS continues to be the predominately used illicit drug in Cambodia [6].

While it is difficult to estimate the proportion of drug users who inject, it is believed to be growing. One study estimated that the injection of drugs had increased from 0.6% of all drug use in 2000 to 10% in 2004 [7]. In 2007, of an estimated 46,300 illicit drug users in Phnom Penh, the vast majority were aged 18–25, most were male; half were regularly using ATS, with only 6.3% reporting regular heroin use while it was estimated that 4.4% of the drug using population in Phnom Penh injected drugs [8]. The NACD estimated that by 2007 there were between 600 to 10,000 injectors in Cambodia [9].

HIV infection was first detected in Cambodia in 1991; since then, transmission has been predominantly heterosexual. Cambodia was one of the countries hardest hit by the HIV epidemic in Asia. With persistent, concerted and targeted efforts to contain the epidemic, the country has been able to significantly reduce the prevalence of HIV from as high as almost 3% to less than 1% [10]. The major prevention and control measures targeted groups at highest risk, especially sex workers and other entertainment workers, the armed forces and mobile populations.

Only in the last 8 years have drug users been recognized as an emerging risk group for HIV. In 2004, HIV among people who inject drugs (PWIDs) was estimated to be between 14% and 31%, compared with between 3% and 18% among non-injecting drug users (non-IDU). In one study in 2007, 24.4% of PWIDs were infected with HIV, compared with 1.1% of non-IDU [10]. One survey reported that the risk factors for continued transmission of HIV among PWIDs and from them to their sexual partners included PWIDs having multiple sex partners, high rates of unsafe sex (40% not regularly using condoms), the selling of blood, and low awareness of HIV transmission – with 47% of PWIDs sharing injecting equipment [11].

The increasing drug availability and use in Cambodia has resulted in the proliferation and use of compulsory drug treatment centers [12], but in 2003 the Government of Cambodia officially began to recognise that harm reduction was an essential approach to preventing HIV among people who used drugs and their sexual partners. Since then, several programs aiming to control and prevent HIV among drug users have been implemented in Cambodia, mostly in the capital, Phnom Penh. However, there have been ongoing tensions between law enforcement and harm reduction actors, despite several advocacy efforts targeting law enforcement. This study attempts to better understand the implementation of harm reduction in Cambodia and how the policy environment and harm reduction program implementation has intersected with the role of law enforcement officials in Cambodia.

## Methods

This research was conducted as part of the Law Enforcement and Harm Reduction at the Nossal Institute (LEHRN) regional project, which looked at the impact of harm reduction on law enforcement policy and practice in Cambodia, Laos and Vietnam. This qualitative research consisted of a background literature review supported by in-depth interviews with key informants. The background document review examined government laws, policies, guidelines, reports, newspaper articles and government announcements related to harm reduction. We conducted 21 in-depth interviews with key informants from law enforcement and health agencies, development partners, NGOs and community representatives. Participants were selected from both a policy and implementation level.

The data were analyzed using thematic analysis. The qualitative computer program Nvivo was used to assist in the thematic coding of interviews. Many themes were explored during the analysis including: interactions between harm reduction programs and law enforcement officials, attitudes of police and the community towards harm reduction, positive and negative influences on harm reduction program implementation, the drivers of police interactions with harm reduction programs and suggestions about what was required to improve the implementation of harm reduction in Cambodia.

## Results

### Evolution of harm reduction in Cambodia

In 2003, against a backdrop of rising HIV prevalence among injecting drugs users and an international community advocating for the need to implement a harm reduction response, the Government of Cambodia developed a policy for mounting a strategic response to harm reduction [13]. An enabling environment for harm reduction began to slowly emerge. High-level political statements began to use language that indicated the government was beginning to conceptualize drug use as a health issue rather than a criminal issue and these statements provided a platform for harm reduction initiatives to move forward. In 2003, Prime Minister Hun Sen stated:

"“In accordance with the drug control law of Cambodia, drug addicted people must have received consultation, treatment and rehabilitation rather than being taken to court. Drug addicted people badly need health support and support from society rather than leaving them as outlawed people of society.”"

Ongoing sustained advocacy efforts, especially of key people within the UN system in Cambodia, led to the subsequent development and changes in policies. In 2004, these advocacy efforts resulted in a memorandum of understanding (MoU) that was signed between the NACD and National AIDS Authority (NAA) to collaborate in preventing drug-related HIV/AIDS [13]. As one UN official familiar with the development of enabling political environments for harm reduction commented,

"“We worked on advocating with NACD and NCHADS about the benefit of collaboration. In 2004 the NACD and the NAA signed an agreement of cooperation that was the result of the advocacy work by the G22 project and national project with UNODC.”- UN Official"

Building the political infrastructure to support harm reduction resulted in the creation of the position of HIV/AIDS coordinator within the Department of Prevention, Education, and Legislation by the NACD. The MoU also led to the establishment of an Illicit Drug-related HIV and AIDS Working Group (DHAWG) in order to support the integration of HIV/AIDS into the full range of illicit drug-related activities nationwide. DHWAG continues to meet every quarter.

While there is no doubt that the sustained international advocacy was critical in creating political space for harm reduction, key informants indicated that the HIV related donor funding opportunities available to the government if it embraced harm reduction also acted as catalysts for increasing political support. In the effort to secure donor investment in HIV prevention, the government may not have fully comprehended what would be required to be internationally acknowledged as implementing harm reduction. Study tours assisted authorities in realizing that harm reduction strategies were supported by a convincing public health argument and harm reduction began to build strong local advocates.

"“It was a kind of all-or-nothing situation. If we wanted to have the project, we had to accept all its components, which included harm reduction. We were reluctant since we knew nothing about harm reduction. However, after learning the potential benefits through conferences and study visits to Australia, we felt proud that we brought the concept to Cambodia”. - Police officers from the NACD"

Further political support for harm reduction was reflected by the language used in the first Five-year National Plan on Drug Control in Cambodia 2005 to 2010 which explicitly noted that one of the strategies was the ‘reduction of risk caused by drug abuse’ [14]. In the same year, the government approved the first needle and syringe program (NSP) in Phnom Penh.

By 2005 it was clear that, politically at least, harm reduction was integrated into Cambodian national policy. In 2005, the NAA National Strategic Plan for 2006–2010 adopted harm reduction as a guiding principle [15], and in 2008 the first National strategic plan for HIV/AIDS related to illicit drug use (2008–2010) was published. In 2010, the Government launched the methadone maintenance program, set up in Khmer-Soviet hospital in Phnom Penh.

In November 2011, a new Law on the Control of Drugs continued to make explicit legal provision for the delivery of harm reduction services and for the receipt of such services by people who use drugs in Cambodia.

"“…The state shall also ensure the provision of services to reduce harms resulting from drug abuses, of health services and national policies aiming to reduce health, social and economic harms due to drugs on individuals, communities and societies…” - from Article 100 of the law on control of drugs (passed by the National Assembly on November 25, 2011)."

### Challenges in program implementation

In 2005, the NACD granted two local NGOs licenses to operate some components of a harm reduction program including the provision of needles and syringes through community outreach and drop-in centre activities. While the intensive advocacy efforts occurring at the highest political levels led to an enabling policy environment, the same level of advocacy efforts were not being made at the local implementation level. The responsibility for advocacy with the local police and the community was given to the NGOs attempting to implement the first NSPs, which placed undue pressure on nascent programs. A disconnect between policy and implementation began to emerge:

"“There was no advocacy program or leaflet to help people [at the community level] understand. Although the government ordered the NGO to conduct advocacy, the NSP never did so. The NSP did not reach standard implementation practices and when problems occurred, the community complained.” - An officer from the NACD"

Upon reflection, staff from organisations providing harm reduction services believe that there should have been more sustained community advocacy and education. It was noted that while harm reduction programs were trying to operate and establish themselves in the community, there was ongoing anti-drug propaganda. Participants felt that harm reduction advocacy should have been as active and sustained as advocacy and health promotion efforts to raise community awareness about HIV.

"“There is still only a very limited promotion of drug use issues even from a drug prevention level. The main strategy used by the government is to inform people and the general public only about the danger of drug use through the use of posters or advertising on the radio and TV. But, there is no sustainable public information that is giving practical information or information about harm reduction. The fact is that this type of useful information is not given to the grass root levels in the society or even to the people developing policy, because they also don’t seem to have received adequate information about the benefits of harm reduction”. - A staff member from an organisation providing harm reduction services in Cambodia."

It became clear that despite the government of Cambodia approving harm reduction, the law enforcement community operating at the local level were never fully aware of the harm reduction programs or their role in supporting program implementation. Law enforcement officers at the local level were not adequately brought in to the initial planning of the implementation stage. In fact, the first exposure many law enforcement officials had to the harm reduction program was at the implementation launch.

"“Local law enforcement officers invited to the launching workshop of the harm reduction program were surprised to see the strategies and unconvinced they would work, but they said they took it as an order from the boss” - an official from the NACD"

Program implementers also acknowledged that despite the enabling policy environment and the need to have law enforcement on side, there was not enough effort made to bring them on board:

"“Policy is good, but there is a gap between policy and practice because relevant implementing agencies, such as law enforcement officers in Phnom Penh and the province, don’t get invited to attend the monthly DHA meeting. This means they are not involved in discussions on implementation nor are they learning about the new harm reduction policy or strategies”. - Harm reduction program implementer"

In fact, participants interviewed remarked that there was no local level participation of the community nor the police at the implementation level, in either local policy or planning development. This reportedly led to a lack of cooperation from the law enforcement community who perceived that harm reduction program implementation was mostly being driven by the donor community with support of the UN agencies.

"“Policies were written by an external consultant. They cannot understand 100% of the real Cambodian context. Sometimes the way that they designed the intervention does not fit with the Cambodian context” - Harm reduction program staff"

The UN readily acknowledges that more advocacy efforts were required into all levels and agencies considered part of law enforcement in Cambodia.

"“I think the fundamental mistake that we made in the UN system was with the partner development. It was pragmatic to support the NACD because they are on the top of the food chain. The problem was we did not invest efficient enough time in developing awareness with the broader law enforcement community in Cambodia.”- UN Official"

Important components of successful harm reduction programs include the coordination and cooperation of multiple sectors of society. In Cambodia this would include policy makers, the NACD officers and law enforcement officers in the community. It should be expected that all of these sectors understand the harm reduction concept and that all partners could unify, coordinate and collaborate. It was clear early on in the implementation of harm reduction in Cambodia that not only was multisectoral coordination and collaboration lacking, but NGOs providing harm reduction services were not coordinated. This lack of coordination at the NGO level had implications for both service coverage and created negative perceptions of the harm reduction programs by the NACD.

"“There were not clear target areas between {NGO X} and {NGO Y}. Both organisations focus on different target people. NGO X focused on the street children aged less than 25 years, while NGO Y focused on people aged over 25 years. They still fight in the same location for distribution and collection of syringes”. - An officer from the NACD"

#### Positive strategies that assisted harm reduction implementation

Participants in this study noted several strategies that had a positive effect on program implementation including the provision of harm reduction training to local police, law enforcement officers, local authorities and communities. Training and sensitisation efforts reportedly led to better communication and collaboration between law enforcement officers and HR implementers. These efforts resulted in the community having a better understanding of harm reduction concepts and slowly changing attitudes of both community and the police towards harm reduction actors and programs and also towards drug users.

"“Police used to hate the outreach staff from {named NGO} because they helped drug users, who we considered as bad people. After attending harm reduction training, police began to take pity on the people working with drug users and decided to cooperate more. Now police understand the role of harm reduction and refer drug users to services”. - Harm reduction implementer"

As community relations improved and communication between the community and programs improved, both community and programs were able to collectively improve the enabling environment for harm reduction services.

"“Before, the community complained about increased crimes, improper needles/syringes disposal, and public order disturbances. Nowadays when the NSP has been implemented the IDUs do not throw away needles and syringes because of encouragement and awareness from the people working with the NSP. Some people in the community even volunteer to help collect needles and syringes”. - Harm reduction program implementer"

Harm reduction implementation also improved when the implementers regularly visited the local police who worked in the district as well as the other local government officials. Furthermore, keeping good relationships between the program implementers and high-ranking government officials was equally important.

"“{Named NGO} has a regular visit to the local police, the official worker and the local social affair officer. We also work regularly on keeping regular contact to the people in high levels of authority. That is the key element; it is not just to go to the people that have the problems, it’s to communicate with everyone”. - Harm reduction program implementer"

#### *Factors that related to negative program implementation experience*

One of the NGOs that had been granted a license to implement NSPs did not have its license renewed by the NACD. While the exact reasons for this remain unclear, perceived issues associated with the program management were thought to play a part. The police received complaints from local communities about increased crimes, improper needles/syringes disposal, and public order disturbances. The police also suggested that drug dealing around the drop-in center gave the police an uneasy feeling about the program that resulted in a lack of trust, communication and cooperation. It was also believed that international people associated with the program were not sensitive enough towards local Khmer customs and culture with regard to the need for formality when interacting with government officials.

"“The community asked the police to close {Named NGO} because there were syringes with blood everywhere. The syringe collectors could not collect the syringes properly and that could have led to HIV transmission. Therefore, the impact of the program appeared to be more negative than positive. This showed that inappropriate implementation of harm reduction can have a bad impact”. - Local police officer familiar with the issue"

#### The role of the NACD in harm reduction implementation

Harm reduction in Cambodia ostensibly falls under the responsibility of the NACD. The NACD is meant to be the national management and coordination mechanism for all drug related issues in Cambodia. The NACD is meant to coordinate the flow of information about harm reduction both horizontally and vertically but believes that its role in communicating information about harm reduction to other police agencies is somewhat compromised because it appears their harm reduction mandate contradicts that of the Anti-Drug Department.

"“The Anti-Drug Department’s role is to crack down on drugs, arrest drug users and drug dealers. So, it is difficult to talk about harm reduction concepts with the Anti-Drug Department”. -An officer at NACD"

The NACD however sees itself as the nominal watchdog for harm reduction implementation in Cambodia and in addition to controlling the licenses for harm reduction programming, attempts to monitor the NSPs and their ability to collect used needles.

"“The NACD should control needle and syringe programs (NSPs), how needles and syringes are distributed and collected. For example, 10,000 needles and syringes were distributed, but if there were only 2,000 needles and syringes collected, where are the 8000 needles and syringes? We need to know whether they sold them or injected with them and then disposed them everywhere in the communities.”- Official from the NACD"

Despite having the legal mandate to monitor harm reduction programs, some observers believe the NACD does not have the technical capacity to manage the whole spectrum of drug related issues related to health and HIV including evidenced-based treatment. The NACD even acknowledges that people within their own department do not support harm reduction.

"“Even though the NACD launched the harm reduction policy in 2009, police still don’t understand the concept” - an official at NACD"

### Perceptions of harm reduction across society in Cambodia

Harm reduction concepts have not been well understood by the general public in Cambodia. While the concept itself is a relatively new concept for managing drug issues in Cambodia, there is actually no single Khmer word that defines the concept. Harm reduction refers to a set of programs and approaches aiming to reduce harms resulting from drug abuse. At the policy level, this concept is fairly well understood. However, at local level where English is not spoken, NACD officials refer to the Needle and Syringe program as harm reduction even though they acknowledge that harm reduction entails much more.

"“When we talk about harm reduction, people always refer to needles and syringe. In fact, harm reduction is about many things” - Official from the NACD"

#### Perceptions of drug users and harm reduction

The implementation of harm reduction in Cambodia has been negatively affected by the ongoing negative community perceptions and attitudes towards drug users. In Cambodia, drug users are often thought to be associated with crimes such as theft and violence. Police officers report that many drug users are believed to be involved in distributing drugs and they use this perception to justify ongoing arrest of drug users.

"“It’s difficult to distinguish between drug users and drug distributors. They are doing both”. - Local law enforcement official"

"“7 out of 10 drug users are also drug sellers. They must be arrested.” - Local law enforcement official."

As the harm reduction concept is generally not that well understood, people across many levels of Cambodian society have a negative attitude towards harm reduction. At a policy level, despite the fairly good understanding of the harm reduction concept, people are still in doubt of its applicability in the Cambodian context. A lack of locally generated evidence of the success of harm reduction in Cambodia continues to hamper ongoing advocacy efforts across the community.

"“At the beginning, we were proud to be the first to bring the program into Cambodia. Now we fear that we might be bringing an inappropriate concept into this country.” - Complaint officers at NACD"

"“There is no evidence of NSP, although the NSP has been implemented since 2005. We have not had evidence based on NSP in our country. We only took two or three cases of evidence from other countries. I have been angry for a long time with NSP, there has been no evidence base although it has been implemented for a long time. Differently, in Taiwan after only one year of implementation, evidenced base is available now.” - An officer at NACD"

The doubts are more pronounced at the local police and community level. Despite some support developed among local police towards people implementing harm reduction programs, simply collecting inappropriately disposed needles and syringes has not led the communities to believe that the program will make their communities safer.

"“Providing needles and syringes would encourage drug users to continue using drugs”- said a local police officer"

"“Locals say syringe handouts draw criminals” Phnom Penh Post article: [Sept 28, 2009]."

### Law enforcement operational culture and practice

During interviews with key informants it became clear that the police in Cambodia do not perceive their role as including reduction of individual level drug related harm. Police are preoccupied by their role in keeping public order, security and safety in the communities.

"“Police are ordered to crack down on drug users nearly everyday”. - A local level law enforcement official"

Furthermore, the role of police in HIV prevention, treatment, care or support has never been perceived or acknowledged by the local police. Local police actually played down the significance of drug use in HIV infection.

"“NSPs encourage drug users to continue using drugs but not reduce HIV transmission. The actual numbers of HIV infections transmitted by injecting drug is much less than by sexual infection”. - Local law enforcement official"

While the police do not acknowledge their role in HIV prevention, their perceived understanding of the patterns and trends of drug use and the culture of drug using was high. Their knowledge about the cycle of drug dependence and its relation with crime was given as one reason that NSP licenses may not be given out:

"“Injecting drug users inject drugs 3 to 4 times a day, it cost 1200 Riels every time. Often the jobs that drug users do is collecting recycled waste disposal, and they may be stealing something for money as well. If we continued to give licenses for NSPs, we encourages drug users to do illegal work.” - An official from the NACD"

Some key informants acknowledged that while some police officers understand the harm reduction concept, when their boss orders them to solve the problem of drug users, they try to push drug users from their local authority areas in order to keep the area clean. They are essentially ordered from higher level to get rid of drug users as part of their duties. In addition, it is clear that crimes associated with drug use are perceived to be crimes first and health related issues second.

"“When the monitoring comes and the director comes they need to go out and arrest 50 drug users or whatever. They have to do it. It’s their job. It is a very difficult position because you learn to believe in a certain process and then you are told by the supervisor to do something which is completely different”- Local police official"

"“Police understand that drug users are a victim, but when drug users steal bicycles and some properties from local people, polices have to arrest them” - Local police official"

The successful implementation of harm reduction was perceived by police to occur when law enforcement officers turned a blind eye towards the NSP.

"“When outreach staff distributes needles and syringes to DU, my staff and I don’t take any action because there is a licensed program. However, for me I don’t agree with NSPs because it encourages drug users to use more drugs”. - Local police official"

Many participants believe that the local police have a low level of knowledge of harm reduction as a concept and how harm reduction works in practice. Furthermore, they suggest that law enforcement officers are unaware of the public health benefits of harm reduction. This is somewhat supported by the fact that only a very small number of local level Phnom Penh police have ever received training in harm reduction.

"“The belief that harm reduction approaches help reduce HIV transmission among drug users is not shared by people, even at NACD” - An official from NACD"

"“They don’t understand harm reduction well, so they try to arrest drug users rather than consider that drug users are a victim because they think that if they don’t arrest drug users they cannot find drug dealers.” - Harm reduction program implementer"

#### Investing in police training and relationship building

In order to change attitudes of police toward harm reduction approaches, investing in training appears to yield positive results in terms of police attitude and environment for service delivery:

"“I used to hate outreach staff from {Named NGO} because they helped drug users, who we considered as bad people. After attending harm reduction training, I began to take pity on the people working with drug users and decided to cooperate more, now we understand the role of harm reduction and refer drug users to services” - a police officer."

When programs began working actively with police, it has been shown in Cambodia that police can play an important role in getting people in connection with harm reduction programs.

"“{Named NGO} has a good relationship with local police through collaboration with us and when there are street children, using drugs and stealing something, we always refer street children to {Named NGO}”- A local police official"

At the local level drug use is regarded more as a security issue than a social and health issue in Cambodia. From the community perspective, the state of the security of their neighborhood takes precedence over that of HIV prevention, which is viewed as more an issue of individuals. Responding to this expectation, the government has put forwards the ‘commune/village safety policies’ for local law enforcement agencies to implement [16]. The policy defines ‘safe communities’ as those with no thefts/robberies, gambling, drug use, prostitutions or criminal. This has put local law enforcement officers in very difficult dilemma with regards the implementation of harm reduction. In addition, local police in Cambodia are often requested by the parents of drug users in the local community to take them away to the rehabilitation centers.

"“Parents told us to just take their children away, anywhere, because they cause lots of troubles in the families and they have no money to pay for the rehabilitation”. - A local police official"

### Moving forward with harm reduction in Cambodia

"“The issue of injecting drug use and HIV in Cambodia can be compared to a time bomb. If it explodes, we could see a second wave of HIV/AIDS in Cambodia”. - UN Official"

Comprehensive harm reduction programs need to be taken to scale in Cambodia to avert a secondary epidemic of HIV among people who use drugs and their sexual partners. To move forward with harm reduction in Cambodia, it is clear that many structural components associated with both program delivery and with the ongoing tension between the role of programs and the role of police, have to be resolved. It is clear that widespread advocacy, awareness raising and education across multiple sectors and levels of Cambodian society are required. In addition, the coordination of harm reduction programs at the local level requires a much better mechanism that includes local religious actors.

"“HR can work here. It can penetrate into the communities through persons who have decision-making power in the communities. The head of the monks in the communities have great influence on the residences, better than the chief of police in the communities.” - A staff member from a harm reduction implementation program"

Capacity building at the local level on the technical components of harm reduction is crucial component of moving forward with harm reduction in Cambodia.

"“NACD, law enforcement officers, local authorities, communities, and relevant partners should understand harm reduction concepts and practices well. Then society will accept it.” - An official from the NACD"

## Discussion

This study has shed some light on the impact of harm reduction on law enforcement in Cambodia. It also raised some potential ideas for ensuring that harm reduction in Cambodia can move forward in the future. Evidence from many settings around the world suggests that harm reduction is effective in preventing the spread of HIV among drug users and beyond. However, each country has its own particular political, social, cultural and economic context. This particularity requires that harm reduction programs be designed in a way that adapts well to its local context to be successful [17]. The success of the program requires not only enabling policies but also a society that supports the rights of drug users to access services without fear of being arrested or discriminated. In Cambodia, there is much work to be done, as the prevailing attitude towards drug users remains negative [18].

The capacity of implementing agencies to do harm reduction is still very limited. There is no clear or consistent understanding or approach to what constitutes harm reduction or the delivery of such services among organisations that deliver services to people who use drugs in Cambodia. There is a need for an effective coordination mechanism, and for national guidelines, standard operating procedures and protocols on rehabilitation and reintegration of people dependent on drugs for services being provided by NGOs.

The main focus of harm reduction is to prevent HIV transmission among drug users and their sexual partners and the Cambodian government recognises that drug users are an important target group in the fight against the spread HIV. Harm reduction is however mainly handled by the NACD, a body whose primary task is to deal with drug production, trafficking and use. It is difficult for the NACD to, at the same time, promote harm reduction and control drugs while the two concepts are politically, socially and culturally at odds. In addition, the Village Commune Safety Policy directly conflicts with the implementation of harm reduction programs and creates and challenging operational environment for the police who are on one hand being asked by harm reduction implementers to allow harm reduction programs to success while on the other being told to “clean up” the streets.

HIV programs involving other target group such as commercial sex workers face similar challenges, for example, the 100% condom use program has ongoing intervention challenges due to the anti-human trafficking law [19]. The 100% condom program perhaps faces less negative influences from law enforcement compared with those faced by harm reduction programs as sex work is more socially tolerated than drug use, and a higher emphasis has been placed on HIV among sex workers by the HIV/AIDS control agencies. There is strong but perhaps fragile political support for harm reduction. There need to be more concerted efforts to sustain the momentum. In addition, too little attention is paid to local contexts – “local solutions for local contexts”: if it doesn’t work at the community level, it doesn’t matter how good policy and law are.

There has been very limited monitoring or evaluation of harm reduction service delivery and outcomes in Cambodia. Evidence of program effectiveness is essential to influence policy [20]. It is clear that locally generated evidence is essential to supporting advocacy effort. Since harm reduction is context sensitive, evidences of success in other cultural contexts would not be sufficient to sustainably influence policy, attitude and practice here in Cambodia. Local programs have yet to produce convincing evidence to show that harm reduction would do more good than harm to drug users and communities at large.

The researchers acknowledge that there are significant efforts being undertaken by all relevant actors in Cambodia to find solutions that will allow harm reduction programs to succeed within the Village Commune Safety Policy framework. This paper suggests that ongoing collaboration between the Government of Cambodia, its law enforcement agencies, the UN and local and international NGOs is required to find solutions that work “on the ground” in the Cambodian specific context. These collaborative efforts should continue to be aimed at enhancing the ability of law enforcement agencies to be positive collaborators and enablers of HIV prevention programs among all key affected populations in Cambodia, including HIV prevention among and from people who use drugs.

## Competing interests

The authors declare that they have no competing interests.

## Authors’ contributions

KC and SL were responsible for the collection of all primary data in Cambodia and responsible for the analysis and first draft of the manuscript. NT provided assistance with analysis and editing subsequent versions of this manuscript. TM and NC provided technical research guidance during all stages of the research design and data collection as well as providing ongoing assistance with editing the final version. All authors read and approved the final version of this manuscript.
